# Virtual-reality induced nostalgia: Duration and psychological benefits

**DOI:** 10.3758/s13428-025-02851-8

**Published:** 2025-10-16

**Authors:** İrem Yılmaz-Özdemir, Tim Wildschut, Constantine Sedikides, Erich W. Graf

**Affiliations:** 1 School of Psychology, University of Southampton, Southampton, SO17 1BJ UK; 2 Cukurova University, Adana, Turkey

**Keywords:** Nostalgia, Emotion induction, Virtual reality, Psychological benefits

## Abstract

We developed a new nostalgia induction using virtual reality. We compared this virtual reality task (VRT) with the established event reflection task (ERT) in terms of intensity of felt nostalgia and strength of psychological benefits produced by each induction method (Experiment 1) and the durability of these effects over time (Experiment 2). Offering initial validation for the VRT, Experiment 1 revealed that felt nostalgia and psychological benefits were higher in the nostalgia condition than in the control condition, irrespective of the induction method. In Experiment 2, we improved the VRT and measured felt nostalgia and psychological benefits at five time points, separated by 5-min intervals. The augmented VRT produced significantly stronger effects on felt nostalgia than did the ERT, and it retained this advantage over time. Compared to the ERT, the VRT did not produce significantly stronger effects on psychological benefits. Instead, psychological benefits were higher in the nostalgia than control condition at each time point except the final one, irrespective of induction method. Virtual reality environments can induce vivid, durable feelings of nostalgia and ensuing psychological benefits.

## Introduction

Nostalgia, “a sentimental longing or wistful affection for the past” (The New Oxford Dictionary of English, 1998, p. 1266), is a frequently felt (several times a week; Hepper et al., 2021; Wildschut et al., 2006) self-relevant emotion (Sedikides et al., 2025; Van Tilburg et al., 2018). Nostalgia is experienced across cultures (Hepper et al., 2014, 2024) and ages (Hepper et al., 2021; Juhl et al., 2020). When nostalgizing, one brings to mind fond memories from one’s personal past, feels warm and contented, and may sense a tinge of longing for the past (Evans et al., 2021; Hepper et al., 2012). The emotion is steeped in sociality, as the self is almost always surrounded by close others in nostalgic reverie (Juhl & Biskas, 2023; Sedikides & Wildschut, 2019).

Nostalgia conveys a number of psychological benefits (Routledge et al., 2013; Sedikides et al., 2008). The affective signature of nostalgia is predominantly positive (Leunissen, 2023). In experimental studies, nostalgia (compared to control conditions) increases positive (but not negative) affect, and it gives rise to more positive affect than negative affect (i.e., a positivity offset; Leunissen et al., 2021). Also, by bestowing “an endearing luster on past selves” (Davis, 1979, p. 41), nostalgia boosts self-esteem (Holak & Havlena, 1992; Vess et al., 2012). Furthermore, as nostalgia often revolves around social relationships (e.g., parents, children, friends), the emotion bolsters social connectedness and empathy (Sedikides & Wildschut, 2019). Finally, by reviving personally meaningful experiences and landmark life events (e.g., weddings, anniversaries, graduations), nostalgia strengthens self-continuity (i.e., a sense of connection between one’s past and present self; Sedikides & Wildschut, 2025) and imbues life with meaning (Sedikides & Wildschut, 2018).

To study the causal effects of state nostalgia (i.e., transitory, in-the-moment feelings of nostalgia), researchers have experimentally induced the emotion. By far the most frequently used nostalgia induction is the event reflection task (ERT). In the nostalgia condition, participants are given a definition of nostalgia (e.g., “a sentimental longing or wistful affection for the past”) and asked to bring to mind a nostalgic memory from their personal past. They are then prompted to generate keywords that capture the gist of the memory and instructed to write about the nostalgic event for a few minutes. Participants in the control condition are typically instructed to recall an ordinary event from their personal past, list keywords that capture the event, and write about the event (Sedikides et al., 2015a, 2015b; Wildschut & Sedikides, 2025). A meta-analysis of nostalgia experiments showed that 85% of studies induced nostalgia with the ERT (Leunissen et al., 2021; see also Fetterman et al., 2025).

The vivid autobiographical recall on which the ERT relies is particularly effective for inducing emotions with high personal relevance (Joseph et al., 2020), which may explain its prevalence. Yet, methodological diversity is a prerequisite for valid causal inferences, and near-exclusive reliance on a single procedure or instrument renders a literature vulnerable to mono-operation bias (Shadish et al., 2002). Our first objective, then, was to diversify the methodological arsenal by developing and validating a new nostalgia induction. We identified virtual reality (VR) as a promising tool. VR delivers a computer-generated, three-dimensional, artificial environment, viewed via a head-mounted display. It enables immersive experiences in a secure laboratory setting, simulations of complex situations, and a degree of experimental control that can be difficult to achieve in everyday settings (Diemer et al., 2015; Diniz Bernardo et al., 2021). Involvement (i.e., one’s ability to focus on the stimuli that are presented via VR), immersion (i.e., being engaged in the experience), and presence (i.e., a feeling of “being there”) are among the key elements of VR, making it an effective tool for emotion elicitation (Felnhofer et al., 2015). Indeed, researchers have used VR to evoke a range of emotions, including joy, anger, boredom, anxiety, and sadness (Felnhofer et al., 2015), awe (Chirico et al., 2018), fear (Thomson et al., 2019), and angst (Morie, 2006). An effective VR induction of nostalgia would have therapeutic potential. For example, nostalgia confers key benefits to people living with dementia (Ismail et al., 2018), yet their ability to retrieve episodic memories is progressively impaired, rendering inductions that rely on autobiographical recall, like the ERT, less suitable in advanced disease. By presenting immersive, multisensory environments, VR could help people living with dementia to reap the benefits of nostalgia.

Our second objective was to examine the unfolding of nostalgia and its psychological benefits over time. To understand emotions, one has to consider their dynamic nature, including the way in which they unfold over time (Kuppens & Verduyn, 2017). Emotions are complex, dynamic states that can last from only a few seconds to several hours or longer (Verduyn et al., 2015). Despite duration being a fundamental aspect of emotions, there has been no research on the time course of nostalgic episodes and their associated psychological benefits. We filled this gap in Experiment 2.

## Overview

We conducted two experiments. In Experiment 1, we developed a new nostalgia induction using VR, the virtual reality task (VRT). We compared this new induction with the ERT in terms of nostalgic intensity (i.e., the manipulation check) and ensuing psychological benefits. We hypothesized that both the ERT and VRT would successfully induce nostalgia. That is, across induction methods, nostalgic intensity should be higher in the nostalgia than the control condition (H1). We further hypothesized that both inductions would convey psychological benefits, such that positive (but not negative) affect, self-esteem, social connectedness, empathy, self-continuity, and meaning in life would be higher in the nostalgia than control condition (H2). Finally, as VR offers an immersive environment that can be used to create engaging, life-like renderings of nostalgic scenes, we hypothesized that the effect of nostalgia (compared to the control) on felt nostalgia (H3) and psychological benefits (H4) would be stronger in the VRT than ERT.

Based on experience gained in Experiment 1, we revised and strengthened the VRT induction in Experiment 2, and again compared it to the ERT. In Experiment 2, we also addressed, for the first time, the temporal unfolding of nostalgic episodes by assessing felt nostalgia and psychological benefits five times at equally spaced 5-min intervals over a 20-min period. Accordingly, Experiment 2 went beyond Experiment 1 by comparing the ERT and VRT inductions in terms of their capacity to prolong felt nostalgia and its psychological benefits over time. Emotion intensity at onset is positively associated with emotion duration, and the eliciting stimulus plays a critical role in determining emotion intensity (Verduyn, 2021; Waugh et al., 2010). We hypothesized that the VRT (compared to ERT) would enable users to relive and experience past events and places more vividly and intensely, prolonging their felt nostalgia (H5) and attendant psychological benefits (H6). We made data and analysis code available at https://osf.io/tn3pr/?view_only=59f788cbff354c2ab29b5516f2734cee.

## Analysis strategy

To test our hypotheses pertaining to the psychological benefits of nostalgia (i.e., H2, H4, H6), we averaged across ratings of positive affect, self-esteem, social connectedness, empathy, self-continuity, and meaning in life, creating an index. We focused our analyses on this index of psychological benefits for economy of exposition and because doing so enabled us to identify general patterns across the various psychological benefits. We noted when specific results for any given psychological benefit deviated from the general patterns. We assessed negative affect in both experiments but did not include it in the index of psychological benefits, because nostalgia inductions tend not to reduce negative affect (Leunissen et al., 2021); that is, reduced negative affect is not considered a psychological benefit of nostalgia. We report (null) results for negative affect in the Supplemental Material.

## Experiment 1

### Method

#### Participants and design

One hundred and sixty-seven University of Southampton students and staff (104 women, 63 men) completed the experiment (*M*_*a*ge_ = 27.92, *SD*_age_ = 10.45, *Range*_age_ = 18–85). Of these, 112 took part in exchange for course credits. The remaining 55 participants completed the experiment voluntarily. Forty-one percent of participants were British, and 59% had other nationalities. The experiment was approved by the Ethics Committee of the University of Southampton (Reference: 47153).

We induced nostalgia (vs. control) with two induction methods, the ERT and the VRT, resulting in a 2 (nostalgia: nostalgia vs. control) × 2 (induction method: ERT vs. VRT) between-participants design. We randomly assigned participants to conditions: nostalgia-ERT (*n* = 42), control-ERT (*n* = 41), nostalgia-VRT (*n* = 43), control-VRT (*n* = 41). Participants completed the experiment in a laboratory.

#### VR stimuli

**Fig. 1:**
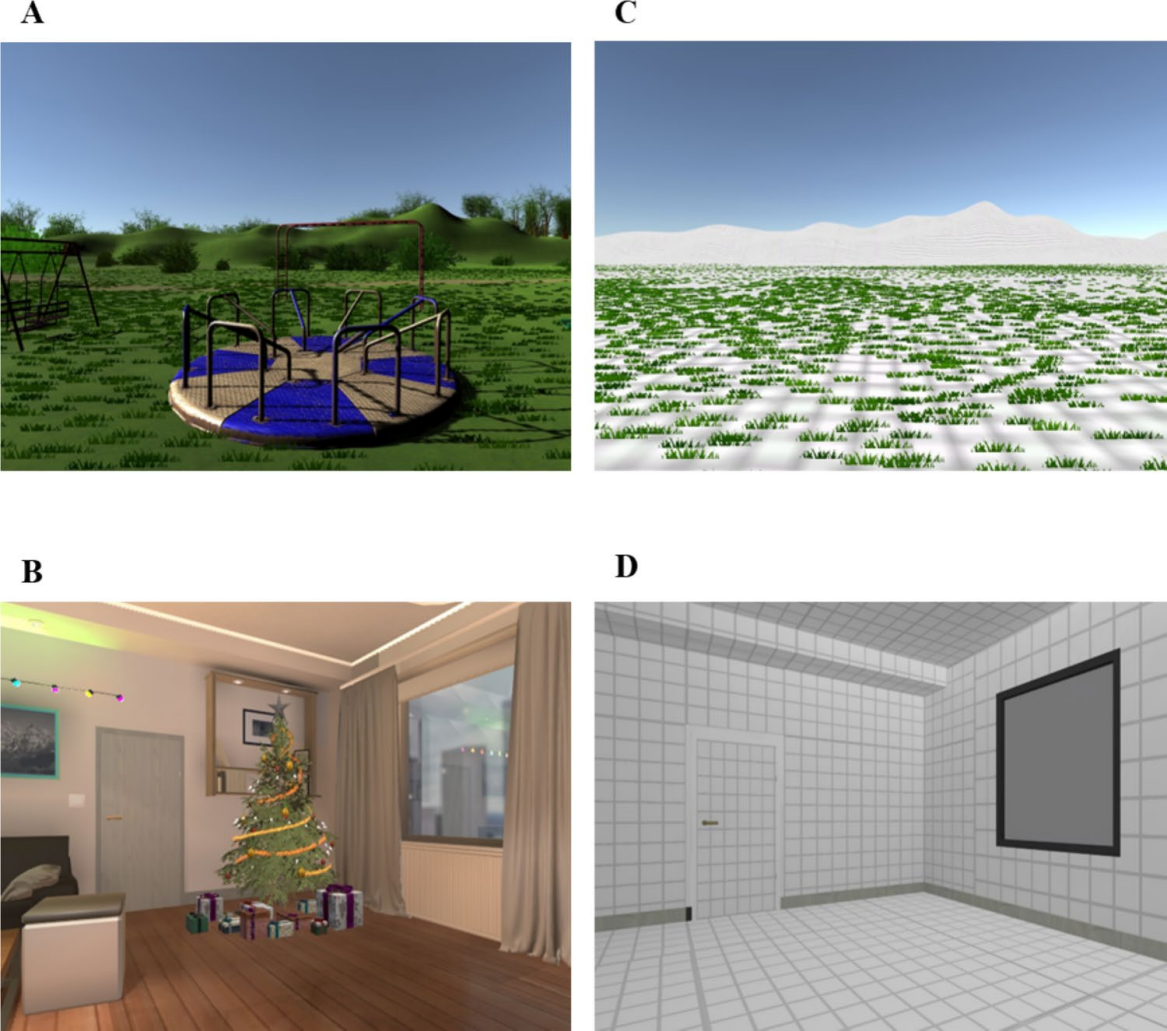
First-person view of nostalgic and control virtual environments in Experiment 1. *Note.* **A** The playground environment. **B** The Christmas environment. **C** The empty outdoor space in the control condition (matching the playground environment). **D** The empty living room in the control condition (matching the Christmas environment)

To develop the VR induction, we first reviewed the literature on the content of nostalgic memories. This literature demonstrates that nostalgic recollections are often associated with specific periods, such as childhood (Batcho, 1995), special events, such as holiday gatherings (Wildschut et al., 2006), and significant others, such as family members and friends (Holak & Havlena, 1992). In addition, to determine the specific themes for the virtual environments, we used NVivo’s word frequency query and created a word cloud (Supplemental Material) from 668 nostalgic narratives collected in previous studies (Cheung et al., 2013, Study 1; Hepper et al., 2021, Study 1; Wildschut et al., 2006, Study 2). Based on this, we created two virtual environments: (1) a Christmas theme consisting of a living room with elaborate Christmas decorations, and (2) a childhood theme, consisting of a playground with a see-saw, swings, and other children’s play equipment. We created control environments for the Christmas and childhood themes, each of which comprised an identical spatial layout without decorations. Figure 1 presents a first-person view of the two nostalgic environments and their matched control environments (for a panoramic view, see Supplemental Material).

We generated the environments using the Unity 3.2f.1 game engine (Unity Technologies, 2018) with task-related components purchased from the Unity Asset Store (https://assetstore.unity.com). We presented the environments via an Oculus Rift VR head-mounted display with 1080 × 1200 resolution per eye (Facebook Inc.). We used a Dell computer with an Intel i7 processor and Windows 10 operating system.

#### Virtual reality task

Before participants in the nostalgia condition put on the VR headset, we showed them a screenshot from each of the two nostalgic environments and asked them to select the one they wished to view. After donning the VR headset, the experimenter read out the definition of nostalgia and instructed participants to recall a nostalgic memory from their past related to the nostalgic environment they had selected and then to describe the event verbally. The instructions were:

According to the Oxford Dictionary, ‘nostalgia’ is defined as a ‘sentimental longing for the past’. Please think of a nostalgic event in your life related to the environment that you are experiencing now. Specifically, try to think of a past event that makes you feel most nostalgic. Bring this nostalgic experience to mind. Immerse yourself in the nostalgic experience. How does it make you feel? For the next 7–10 min, you will speak about the nostalgic event while looking around the environment. When you are ready, you can start describing your memory and how it makes you feel.

We showed participants in the control condition a screenshot from each of the two control environments and asked them to select one for viewing. We then instructed them to bring to mind an ordinary past event that happened outside or at home while looking around the selected virtual environment:

Please think of an ordinary event in your life related to the environment that you are experiencing now. Specifically, try to recall an ordinary memory from your past that happened outside [if the participant selected the control environment matching the layout of a childhood playground] or at home [if the participant selected the control environment matching the layout of a living room]. Bring this ordinary memory to mind and immerse yourself in the ordinary experience. How does it make you feel? For the next 7 to 10 minutes, you will speak about the ordinary event while looking around the environment. When you are ready, you can start describing your memory and how it makes you feel.

Participants in both conditions verbally described their nostalgic or ordinary experience and then listed four keywords to summarize the event.

#### Event reflection task

The ERT is a validated nostalgia induction involving vivid autobiographical writing. The original version of the ERT includes instructions that prompt participants to recall either a nostalgic or ordinary memory from their past (Sedikides et al., 2015a, 2015b). We modified the ERT to better match the VR induction. Specifically, as the VR induction offered participants a choice between a Christmas and a childhood playground environment, we created two matching options within the ERT. In the nostalgia condition, we gave participants a definition of nostalgia (“According to the New Oxford Dictionary of English, nostalgia is defined as a sentimental longing for the past”) and then instructed them to recall a nostalgic event from their past relating to Christmas or their childhood (“Please think of a nostalgic event in your life related to [A] Christmas holiday or [B] your childhood. Bring this nostalgic experience to mind. Immerse yourself in the nostalgic experience. How does it make you feel?”). In the control condition, we instructed participants to bring to mind an ordinary event that happened outside or at home (“Please bring to mind an ordinary event in your life [i.e., an ordinary event that happened outside or at home]. Specifically, try to think of a past event that is ordinary. Bring this ordinary experience to mind. Immerse yourself in the ordinary experience. How does it make you feel?”). Next, we instructed participants in both conditions to list four keywords describing the nostalgic or ordinary event that they recalled, and then to write about that event with vivid and descriptive details.

#### Dependent variables

Following the nostalgia induction (ERT or VRT), we measured the intensity of felt nostalgia with three items (e.g., “Right now, I am having nostalgic feelings”; 1 = *strongly disagree*, 7 = *strongly agree*). We adopted this three-item scale from Wildschut et al. (2006).

Next, we assessed psychological benefits of nostalgia: positive affect (six items; e.g., “happy”), self‐esteem (four items; e.g., “value myself more”), social connectedness (four items; e.g., “connected to loved ones”), empathy (12 items; e.g., “compassionate”), self‐continuity (four items; e.g., “connected with my past”); and meaning (four items; e.g., “life is meaningful”). Items were rated on a six‐point scale (1 = *strongly disagree*, 6 = *strongly agree*) and preceded by the stem “Thinking about this event makes me feel …” We adopted the measures of positive affect, self-esteem, social connectedness, and meaning from Hepper et al. (2012), the measure of empathy from Batson et al. (1987), and the measure of self-continuity from Sedikides et al. (2016).

We present descriptive statistics, scale reliabilities, and correlations among the dependent measures in Table 1. The psychological benefits were positively and significantly correlated. We averaged them to create an overall benefits index (α =.87,* M* = 4.20, *SD* = 0.88). Self-reported nostalgia was positively and significantly correlated with all psychological benefits.

**Table 1:** Correlation matrix for felt nostalgia and psychological benefits in Experiment 1

	*M*	*SD*	α	1	2	3	4	5	6
1. Felt nostalgia	4.76	1.82	.98						
2. Positive affect	4.27	1.31	.86	.53					
3. Self-esteem	4.03	1.26	.92	.23	.55				
4. Social connectedness	4.20	1.52	.90	.64	.56	.35			
5. Empathy	3.92	1.40	.94	.49	.48	.40	.65		
6. Self-continuity	4.34	1.40	.76	.63	.54	.37	.60	.58	
7. Meaning	3.76	1.34	.90	.49	.47	.46	.67	.49	.60

All correlations, *p* <.003

### Results

#### Felt nostalgia

We ran a 2 (nostalgia: nostalgia vs. control) × 2 (induction method: ERT vs. VRT) ANOVA on felt nostalgia. We present descriptive statistics in Table 2 and inferential statistics in Table 3. The analysis revealed a significant main effect of nostalgia (vs. control) on felt nostalgia. Consistent with H1, felt nostalgia was significantly higher in the nostalgia than the control condition. Neither the main effect of the induction method nor the Nostalgia × Induction Method interaction effect was significant. The absence of a significant interaction effect indicates that the VRT induction did not increase felt nostalgia more strongly than the ERT induction, contrary to H3.^1^

**Table 2:** Means and standard deviations for felt nostalgia and psychological benefits by experimental condition in Experiment 1

	VRT/Nostalgia		VRT/Control		ERT/Nostalgia		ERT/Control	
	*M*	*SD*	*M*	*SD*	*M*	*SD*	*M*	*SD*
Felt nostalgia	5.34	1.39	3.85	2.01	5.50	1.17	3.95	1.79
Positive affect	4.34	0.94	4.15	0.98	4.50	0.87	4.09	1.17
Self-esteem	3.63	1.19	4.02	1.01	4.26	1.07	4.21	1.14
Social connectedness	4.65	0.96	3.70	1.48	4.94	0.81	3.49	1.44
Empathy	4.20	0.95	3.73	1.18	4.32	0.74	3.39	1.12
Self-continuity	4.63	0.95	4.06	0.90	4.82	0.80	3.84	1.29
Meaning	4.35	1.16	4.28	1.43	4.96	0.87	4.11	1.21
Benefits index	4.30	0.76	3.99	0.93	4.63	0.57	3.85	1.02

**Table 3:** Inferential statistics from Nostalgia × Induction method ANOVA on felt nostalgia and psychological benefits in Experiment 1

	Nostalgia main effect			Induction method main effect			Nostalgia × Induction Method		
	*F*	*p*	η_p_^2^	*F*	*p*	η_p_^2^	*F*	*p*	η_p_^2^
Felt nostalgia	36.90	<.001	.185	0.28	.598	.002	0.01	.916	.000
Positive affect	3.94	.049	.024	0.12	.735	.001	0.50	.480	.003
Self-esteem	1.03	.312	.006	5.60	.019	.033	1.69	.196	.010
Social connectedness	41.76	<.001	.204	0.05	.826	.000	1.78	.185	.011
Empathy	20.14	<.001	.110	0.52	.471	.003	2.07	.152	.013
Self-continuity	25.09	<.001	.133	0.02	.888	.000	1.73	.191	.010
Meaning	6.25	.013	.037	1.43	.233	.009	4.53	.035	.027
Benefits index	17.72	<.001	.098	0.56	.454	.003	3.24	.074	.020

Degrees of freedom = 1, 163

#### Psychological benefits

We analyzed the benefits of nostalgia in a series of 2 × 2 ANOVAs. We present descriptive statistics in Table 2 and inferential statistics in Table 3. According to H2, psychological benefits would be higher in the nostalgia than control condition. Consistent with this hypothesis, results revealed a significant main effect of nostalgia (vs. control) on the benefits index. Overall, psychological benefits were higher in the nostalgia than the control condition (Table 3). Self-esteem was the only exception (see also Hepper et al., 2024). It is noteworthy that, whereas the nostalgia manipulation did not increase self-esteem, felt nostalgia was positively and significantly correlated with it (*r* =.23, *p* <.003; Table 1). We return to this issue in General discussion.

The main effect of the induction method on the benefits index was not significant. The exception was again self-esteem, with participants in the ERT condition scoring higher than those in the VRT condition (Table 3).

According to H4, the VRT would produce stronger psychological benefits than the ERT induction. That is, H4 anticipates a Nostalgia × Induction Method interaction effect on psychological benefits. Contrary to this hypothesis, the interaction effect on the benefits index was not significant (Table 3). The exception was meaning in life. However, the significant Nostalgia × Induction Method interaction effect on meaning in life did not reflect the hypothesized pattern (Table 2). Specifically, tests of simple nostalgia effects revealed that nostalgia (compared to control) significantly increased meaning when induced with the ERT, *F*(1, 163) = 10.64, *p* =.001, η_p_^2^ =.061, but not when induced with the VRT, *F*(1, 163) = 0.07, *p* =.792, η_p_^2^ =.000. Tests of simple induction method effects showed that, in the nostalgia condition, participants completing the ERT reported higher meaning in life than those completing the VRT, *F*(1, 163) = 5.62, *p* =.019, η_p_^2^ =.033. In the control condition, the difference between ERT and VRT was not significant, *F*(1, 163) = 0.43, *p* =.515, η_p_^2^ =.003.

### Discussion

We obtained support for the effectiveness of the new VRT nostalgia induction. Felt nostalgia (H1) and psychological benefits (H2) were higher in the nostalgia than the control condition. However, contrary to H3 and H4, the VRT induction of nostalgia was not more impactful than the widely used ERT. To be precise, the effect of nostalgia (compared to control) on felt nostalgia and psychological functions was not qualified by the induction method.

We also found a main effect of induction method on self-esteem, with participants who completed the ERT reporting higher self-esteem than those who completed the VRT (irrespective of whether they were in the nostalgia or control condition). In addition, participants in the nostalgia condition (but not those in the control condition) reported higher meaning in life when completing the ERT than when completing the VRT. A reason why the ERT may have elicited higher self-esteem and (in the nostalgia condition) meaning in life than the VRT is that the former task is idiographic (i.e., elicits memories that are unique to a given individual), whereas the latter is nomothetic (i.e., highlights common experiences that are shared by individuals in a particular cohort or culture, such as celebrating Christmas) and thus potentially less self-relevant.

The key implication of Experiment 1, then, is that the VRT is effective but leaves room for improvement. We therefore strengthened the VRT in Experiment 2 by adding a third environment (i.e., birthday scene) and creating more dynamic, multisensory scenes through the addition of human-like avatars and sound.

## Experiment 2

The key objective of Experiment 2 was to replicate and extend Experiment 1 by examining the efficacy of the strengthened VRT, once again comparing it with the ERT. We tested the same hypotheses as in Experiment 1 (H1–H4). In addition, we assessed felt nostalgia and psychological benefits five times at equally-spaced 5-min intervals over a 20-min period to test if the effects of nostalgia (vs. control) on felt nostalgia (H5) and psychological benefits (H6) are more durable over time when the emotion is induced with the VRT (than ERT).

### Method

#### Participants and design

Two hundred and forty-eight University of Southampton students and staff (169 women, 76 men, 2 other, 1 preferred not to say) completed the experiment (*M*_age_ = 24.31 years, *SD*_age_ = 7.81, *Range*_age_ = 18–70). Students took part in exchange for course credits. We remunerated staff with £5 ($6,64) per hour. Fifty-six percent of participants stated that their first language is English. The experiment was approved by the Ethics Committee of the University of Southampton (Reference: 47153.A5).

The design of Experiment 2 comprised two between-participants variables and one within-participants variable. As in Experiment 1, the between-participants variables were nostalgia (nostalgia vs. control) and induction method (ERT vs. VRT). We randomly assigned participants to one of the four resultant experimental conditions: nostalgia-ERT, *n* = 62; control-ERT, *n* = 62; nostalgia-VRT, *n* = 62; control-VRT, *n* = 62. The within-participants variable was time (Time 1–5). Following the nostalgia induction (ERT or VRT), we assessed felt nostalgia and psychological benefits (i.e., positive affect, self-esteem, social connectedness, empathy, self-continuity, meaning) at five time points. The five time points were separated by four equally spaced 5-min intervals. Time 1 started immediately after the nostalgia induction. We used a filler task to occupy participants during the 5-min intervals between time points. To enable precise timing between measurements, we integrated bespoke JavaScript elements into Qualtrics, which directed participants to a separate Qualtrics webpage every five minutes to complete the assessments. Upon completing the assessments, participants were automatically directed to a filler task. It comprised 37 items, including anagrams (e.g., “Identify the anagram: ‘Salad Lover’—Which city could it be?”), attentional tasks (e.g., “Describe the stages of cooking pasta”), basic mathematical problems (e.g., “200 – (96/4) =?”), and multiple-choice questions (e.g., “Which is a synonym of ‘Empirical’? – ‘Debatable,’ ‘Friendly,’ ‘Provable,’ ‘Murky’”). We presented filler items in a randomized order.

#### VR stimuli

In Experiment 1, we used two virtual environments: (1) a Christmas theme consisting of a living room with Christmas decorations, and (2) a childhood theme, consisting of a children’s playground. We improved these environments in Experiment 2 and added a third environment: (3) a birthday theme, consisting of a living room with elaborate birthday decorations (e.g., birthday cake, presents, balloons). We added this third theme to give participants more choices and thereby increase the likelihood that one of the environments would elicit high levels of nostalgia. The decision to add the birthday scene was informed by the content analysis of nostalgic narratives (see Experiment 1). Additionally, the birthday theme could be readily implemented in a virtual environment, making it a practical and engaging option.

**Fig. 2:**
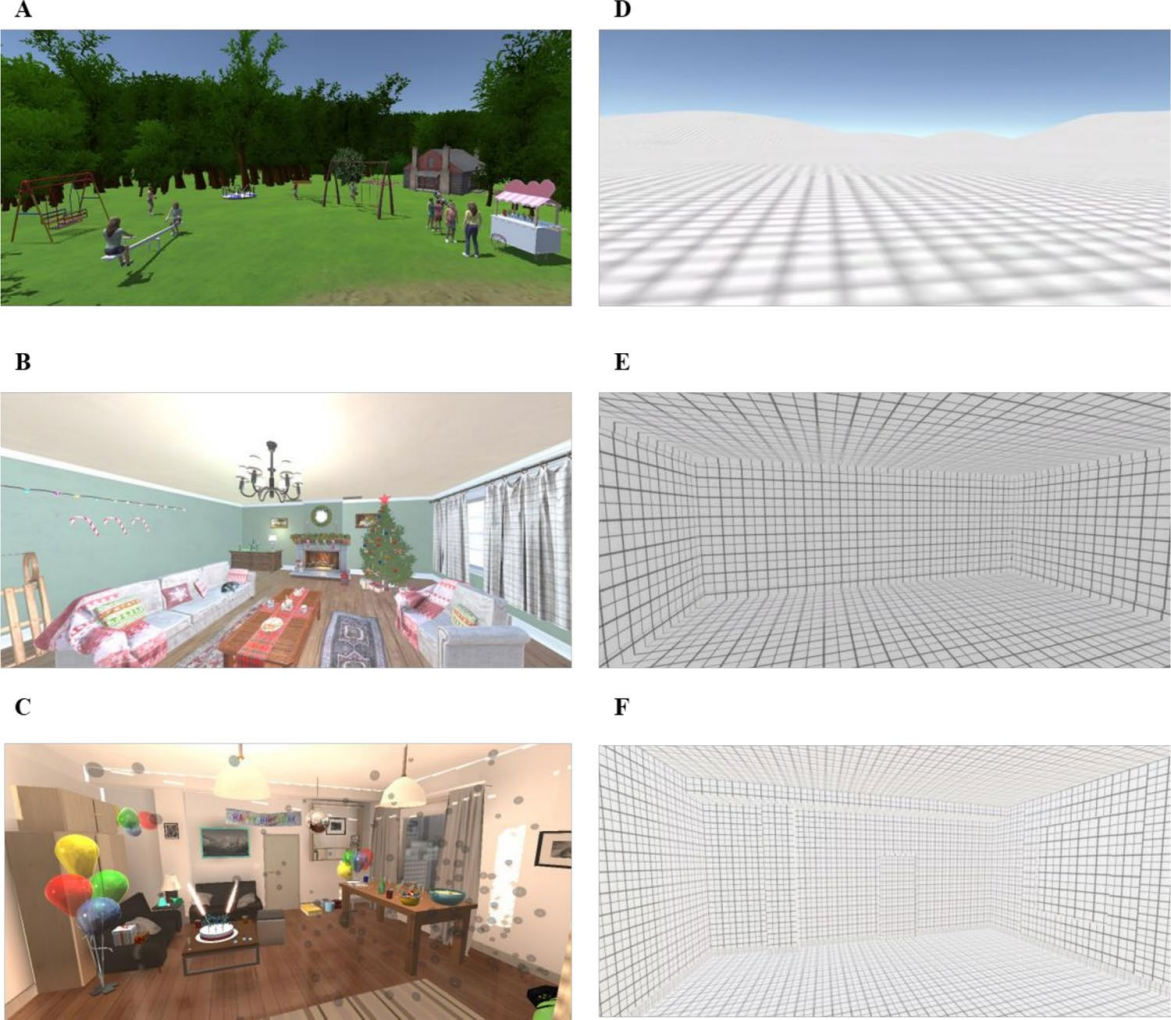
First-person view of nostalgic and control virtual environments in Experiment 2. *Note.* **A** The improved playground environment. **B** The improved Christmas environment. **C** The birthday environment. **D** The empty outdoor space in the control condition (matching the playground environment). **E** The empty living room in the control condition (matching the Christmas environment). **F** The empty living room in the control condition (matching the birthday environment)

We further strengthened the VRT by creating a new Christmas environment with enhanced resolution and more decorations, adding children’s play equipment and human-like avatars to the playground environment to increase its resemblance to real life, and adding scene-related sounds to all three environments. For the Christmas and birthday environments, we used several music clips and joined them together using Audacity (https://www.audacityteam.org/) audio editor. For the playground environment, we added an ambient park sound (i.e., birdsong, voices) that was looped continuously. In the control condition, we presented blank virtual environments with identical dimensions (Baños et al., 2008; Diemer et al., 2015). We presented the environments via an Oculus Rift VR head-mounted display with 1080 × 1200 resolution per eye (Facebook Inc.). We used a Dell computer with an Intel i7 processor and Windows 10 operating system. We present in Fig. 2 a first-person view of the three nostalgic environments and their matched control environments (for a panoramic view, see Supplemental Material).

#### Nostalgia inductions

Instructions for the VRT and ERT were essentially identical to those used in Experiment 1, with minor changes in wording (Supplemental Material). Following the addition of the birthday environment (and matching control environment), participants completing the VRT selected one of three environments. Participants completing the ERT were instructed to recall “a nostalgic event in your life related to either (1) a Christmas holiday, (2) your childhood, or (3) one of your birthdays” (in the nostalgia condition) or to “bring to mind an ordinary event that happened outside or at home” (in the control condition).

#### Dependent variables

Following the nostalgia induction (ERT or VRT), we assessed felt nostalgia and psychological benefits of nostalgia at five time points, using the same measures as in Experiment 1. We report descriptive statistics and scale reliabilities in for each measure at each time point in Supplemental Materials. Average scale reliability was .92 (range =.77 to.98).

The psychological benefits were positively and significantly intercorrelated. To illustrate, we averaged participants’ ratings of psychological benefits across time points and presented the correlations among these average ratings in Table 4 (we also examined correlations among psychological benefits within each time point; all were positive and significant, *p*s <.001.) Within each time point, we averaged the psychological benefits to create an overall index. We further show in Table 4 that, averaged across time points, self-reported nostalgia was positively and significantly correlated with all psychological benefits. (Felt nostalgia was also positively and significantly correlated with all psychological functions within each time point, *p*s <.003.).

**Table 4:** Correlation matrix for felt nostalgia and psychological benefits averaged across time points in Experiment 2

	1	2	3	4	5	6
1. Felt nostalgia						
2. Positive affect	.39					
3. Self-esteem	.37	.71				
4. Social connectedness	.37	.64	.57			
5. Empathy	.43	.57	.46	.73		
6. Self-continuity	.41	.65	.67	.76	.67	
7. Meaning	.35	.65	.65	.66	.55	.74

All correlations, *p* <.001

### Results

We entered felt nostalgia and psychological benefits as dependent variables in 2 (nostalgia: nostalgia vs. control) × 2 (induction method: ERT vs. VRT) × 5 (time: Time 1–5) mixed ANOVAs. We report descriptive statistics in Table 5 and inferential statistics in Tables 6, 7, 8. We first present between-participants effects of nostalgia and induction method, that is, effects averaged across time points. Next, we present results involving the within-participant effect of time, that is, effects describing changes over time (as well as how these temporal changes were moderated by nostalgia and induction method). We partitioned the effect of time with two polynomial contrasts: the linear trajectory over time and the quadratic trajectory over time (Table 8). We do not report the higher-order cubic and quartic trajectories, because they were sporadic and lacked a substantive interpretation.

**Table 5:** Means and standard deviations for felt nostalgia and psychological benefits by experimental condition and time point in experiment 2

	VRT/Nostalgia			VRT/Control		ERT/Nostalgia		ERT/Control	
	*M*		*SD*	*M*	*SD*	*M*	*SD*	*M*	*SD*
Time 1									
Felt nostalgia	5.68		1.22	3.32	1.74	5.68	1.07	4.12	1.67
Positive affect	4.25		1.01	3.94	0.98	4.18	0.82	3.86	1.24
Self-esteem	3.73		1.13	3.77	1.21	3.88	1.07	3.96	1.15
Social connectedness	4.46		1.19	3.29	1.35	4.58	1.02	3.38	1.50
Empathy	4.22		0.93	3.31	1.02	4.06	0.87	3.44	1.18
Self-continuity	4.55		0.81	3.77	1.20	4.80	0.75	3.76	1.22
Meaning	4.22		1.26	4.05	1.20	4.33	1.20	4.27	1.44
Benefits index	4.24		0.81	3.69	0.85	4.30	0.72	3.78	1.04
Time 2									
Felt nostalgia	3.58		1.70	2.36	1.37	2.96	1.76	2.50	1.33
Positive affect	3.86		0.95	3.45	0.84	3.70	1.00	3.60	1.10
Self-esteem	3.60		1.17	3.24	1.20	3.62	1.16	3.71	1.20
Social connectedness	3.94		1.23	2.91	1.32	3.93	1.26	3.22	1.57
Empathy	3.76		1.00	2.98	1.02	3.70	1.09	3.33	1.16
Self-continuity	3.98		0.95	3.26	1.23	4.08	1.26	3.70	1.15
Meaning	3.94		1.15	3.35	1.34	3.75	1.49	3.97	1.45
Benefits index	3.85		0.85	3.20	0.91	3.80	1.04	3.59	1.03
Time 3									
Felt nostalgia	3.16		1.58	2.03	1.29	2.59	1.72	2.49	1.44
Positive affect	3.66		1.02	3.22	0.96	3.59	0.97	3.44	1.24
Self-esteem	3.43		1.13	3.09	1.23	3.50	1.16	3.62	1.24
Social connectedness	3.77		1.19	2.87	1.34	3.83	1.23	3.13	1.62
Empathy	3.51		1.09	2.91	1.10	3.56	1.06	3.22	1.32
Self-continuity	3.75		1.05	3.25	1.34	4.02	1.26	3.56	1.24
Meaning	3.72		1.31	3.27	1.47	3.82	1.46	3.83	1.59
Benefits index	3.64		0.95	3.10	1.00	3.72	1.01	3.47	1.13
Time 4									
Felt nostalgia		2.47	1.51	1.82	1.14	2.48	1.74	2.05	1.37
Positive affect		3.34	1.13	3.27	1.12	3.30	1.14	3.33	1.33
Self-esteem		3.36	1.13	3.08	1.35	3.31	1.35	3.44	1.35
Social connectedness		3.64	1.31	2.77	1.40	3.72	1.36	3.09	1.61
Empathy		3.36	1.16	2.83	1.13	3.37	1.14	3.14	1.38
Self-continuity		3.51	1.06	3.04	1.40	3.92	1.27	3.37	1.42
Meaning		3.60	1.21	3.16	1.53	3.65	1.46	3.84	1.47
Benefits index		3.47	0.99	3.02	1.12	3.54	1.13	3.37	1.18
Time 5									
Felt nostalgia		2.06	1.35	1.76	1.14	2.42	1.80	2.42	1.52
Positive affect		3.20	1.23	3.14	1.08	3.24	1.21	3.43	1.27
Self-esteem		3.17	1.27	2.93	1.30	3.40	1.37	3.70	1.40
Social connectedness		3.54	1.35	2.78	1.36	3.68	1.35	3.16	1.62
Empathy		3.19	1.19	2.76	1.12	3.39	1.23	3.03	1.28
Self-continuity		3.43	1.22	2.92	1.40	3.82	1.41	3.42	1.25
Meaning		3.38	1.30	3.15	1.50	3.62	1.52	3.85	1.50
Benefits index		3.32	1.10	2.95	1.07	3.53	1.20	3.43	1.14
Average across time points									
Felt nostalgia		3.39	1.10	2.26	0.93	3.23	1.31	2.76	1.04
Positive affect		3.66	0.92	3.40	0.82	3.60	0.89	3.53	1.11
Self-esteem		3.46	0.99	3.22	1.09	3.54	1.10	3.69	1.13
Social connectedness		3.87	1.03	2.92	1.17	3.95	1.13	3.20	1.49
Empathy		3.61	0.91	2.96	0.96	3.61	0.99	3.23	1.20
Self-continuity		3.84	0.81	3.25	1.14	4.13	1.07	3.56	1.12
Meaning		3.77	1.01	3.40	1.27	3.83	1.34	3.95	1.38
Benefits index		3.70	0.82	3.19	0.88	3.78	0.96	3.53	1.04

**Table 6:** Inferential statistics from Nostalgia × Induction Method × Time ANOVA on felt nostalgia and psychological benefits: between-participants effects in Experiment 2

	Nostalgia main effect			Induction method main effect			Nostalgia × Induction Method		
	*F*	*p*	η_p_^2^	*F*	*p*	η_p_^2^	*F*	*p*	η_p_^2^
Felt nostalgia	32.41	<.001	.117	1.50	.222	.006	5.72	.018	.023
Positive affect	1.91	.169	.008	0.09	.770	.000	0.64	.425	.003
Self-esteem	0.12	.735	.000	4.02	.046	.016	1.89	.170	.008
Social connectedness	30.00	<.001	.109	1.28	.258	.005	0.39	.531	.002
Empathy	15.87	<.001	.061	1.18	.279	.005	1.09	.297	.004
Self-continuity	19.19	<.001	.073	5.12	.025	.021	0.02	.901	.000
Meaning	0.64	.425	.003	3.69	.056	.105	2.40	.123	.010
Benefits index	10.48	.001	.041	3.05	.082	.012	1.22	.270	.005

Degrees of freedom = 1, 244

**Table 7:** Inferential statistics from Nostalgia × Induction Method × Time ANOVA on felt nostalgia and psychological benefits: Within-participants effects in Experiment 2

	Time main effect			Nostalgia × Time			Induction Method × Time			Nostalgia × Induction Method × Time		
	*F*	*p*	η_p_^2^	*F*	*p*	η_p_^2^	*F*	*p*	η_p_^2^	*F*	*p*	η_p_^2^
Felt nostalgia	206.71	<.001	.459	23.31	<.001	.087	4.65	.001	.019	1.03	.389	.004
Positive affect	67.33	<.001	.216	4.84	<.001	.019	1.36	.247	.006	0.78	.539	.003
Self-esteem	27.94	<.001	.103	1.11	.352	.005	2.76	.027	.011	1.43	.221	.006
Social connectedness	35.27	<.001	.126	5.54	<.001	.022	0.42	.794	.002	0.54	.707	.002
Empathy	58.33	<.001	.193	5.25	<.001	.021	1.89	.110	.008	0.77	.545	.003
Self-continuity	56.86	<.001	.189	4.76	<.001	.019	1.98	.118	.008	1.64	.162	.007
Meaning	43.61	<.001	.152	0.93	.446	.004	1.99	.095	.008	2.35	.052	.010
Benefits index	83.46	<.001	.255	3.65	.006	.015	2.54	.039	.010	1.52	.195	.006

Degrees of freedom = 4, 976

**Table 8:** Inferential statistics from Nostalgia × Induction Method × Time ANOVA on felt nostalgia and psychological benefits: Linear and quadratic trajectories in Experiment 2

		Trajectory main effect			Nostalgia × Trajectory			Induction Method × Trajectory		
	Trajectory	*F*	*p*	η_p_^2^	*F*	*p*	η_p_^2^	*F*	*p*	η_p_^2^
Felt nostalgia	Linear	341.61	<.001	.583	43.30	<.001	.151	1.23	.269	.005
	Quadratic	147.40	<.001	.380	9.04	.003	.036	11.31	<.001	.044
Positive affect	Linear	125.49	<.001	.390	8.11	.005	.032	2.15	.144	.009
	Quadratic	25.31	<.001	.094	1.54	.215	.006	0.01	.943	.001
Self-esteem	Linear	51.69	<.001	.175	0.00	.978	.000	2.55	.112	.010
	Quadratic	16.72	<.001	.064	4.48	.035	.018	1.29	.257	.005
Social connectedness	Linear	53.43	<.001	.180	12.14	<.001	.047	1.01	.316	.004
	Quadratic	25.75	<.001	.095	1.75	.187	.007	0.03	.854	.000
Empathy	Linear	107.77	<.001	.306	9.08	.003	.036	2.87	.092	.012
	Quadratic	18.29	<.001	.070	3.31	.070	.013	0.87	.352	.004
Self-continuity	Linear	113.39	<.001	.317	6.82	.010	.027	4.24	.041	.017
	Quadratic	21.01	<.001	.079	5.34	.022	.021	0.09	.769	.000
Meaning	Linear	79.15	<.001	.245	0.63	.428	.003	4.33	.038	.017
	Quadratic	27.05	<.001	.100	2.74	.099	.011	0.01	.932	.000
Benefits index	Linear	67.30	<.001	.215	6.65	.011	.027	4.16	.042	.017
	Quadratic	44.92	<.001	.156	0.01	.937	.000	0.01	.914	.000

Degrees of freedom = 1, 244

#### Felt nostalgia

##### Between-participant effects: Effects averaged across time points

**Fig. 3:**
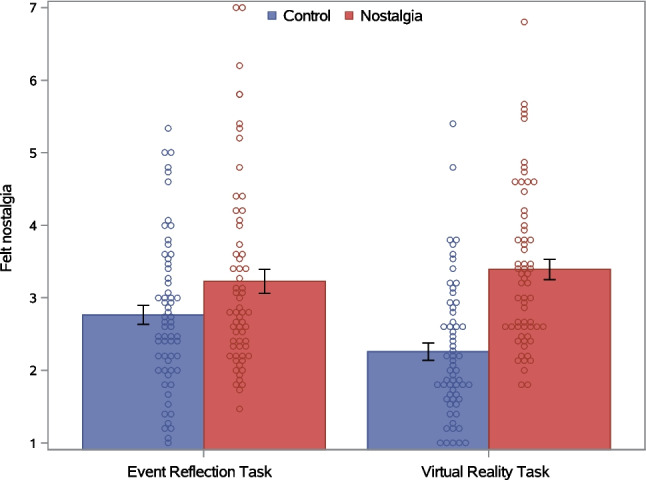
Felt nostalgia as a function of nostalgia (vs. control) and induction method in Experiment 2. *Note. Error bars* represent standard errors

Consistent with H1, a significant main effect of nostalgia (vs. control) indicated that felt nostalgia was significantly higher in the nostalgia than the control condition. The main effect of the induction type was not significant. Importantly, the Nostalgia × Induction Method interaction was significant (Table 6). We depict this interaction effect in Fig. 3.

Consistent with H3, tests of simple effects revealed that, for participants completing the VRT, the nostalgia induction significantly increased felt nostalgia, *F*(1, 244) = 32.68, *p* <.001, η_p_^2^ =.118. For participants completing the ERT, the nostalgia induction also increased felt nostalgia, *F*(1, 244) = 5.45, *p* =.020, η_p_^2^ =.022, but this simple effect was smaller than in the VRT (as indicated by the Nostalgia × Induction Method interaction). Tests of simple induction method effects revealed that, in the nostalgia condition, felt nostalgia did not differ significantly between the VRT and ERT, *F*(1, 244) = 0.68, *p* =.410, η_p_^2^ =.003. In the control condition, participants felt less nostalgic in the VRT than ERT, *F*(1, 244) = 6.54, *p* =.011, η_p_^2^ =.026. Thus, the greater effectiveness of the VRT (compared to ERT) was primarily attributable to lower felt nostalgia in the control–VRT than control–ERT condition.

##### Within-participant effects: Changes over time

**Fig. 4:**
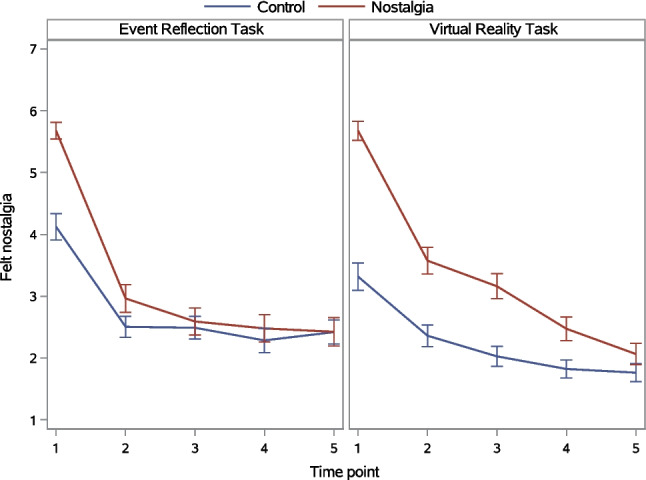
Felt nostalgia as a function of nostalgia, induction method, and time in Experiment 2. *Note. Error bars* represent standard errors

We depict in Fig. 4 changes in felt nostalgia over time as a function of the nostalgia and induction method manipulations. A significant main effect of time indicated that felt nostalgia differed between time points. This time main effect was qualified by significant Nostalgia × Time and Induction Method × Time interactions (Table 7).

We first probed the Nostalgia × Time interaction. Nostalgia (vs. control) interacted with both the linear and quadratic trajectory over time (Table 8). We focused on the interaction between nostalgia (vs. control) and the higher-order quadratic trajectory (because it qualifies the lower-order linear trajectory). Tests of simple quadratic trajectories revealed that this trajectory was significant in both the control condition, *F*(1, 244) = 41.71, *p* <.001, η_p_^2^ =.146, and nostalgia condition, *F*(1, 244) = 114.73, *p* <.001, η_p_^2^ =.320, but was stronger in the latter than the former condition. In both conditions, but particularly in the nostalgia condition, felt nostalgia declined over time, with this drop being most pronounced between Time 1 and Time 2 and leveling off over subsequent time points. Tests of simple nostalgia (vs. control) effects showed this effect was significant, but became progressively smaller, at each of the first four time points: Time 1, *F*(1, 244) = 112.84, *p* <.001, η_p_^2^ =.308; Time 2, *F*(1, 244) = 17.975, *p* <.001, η_p_^2^ =.067; Time 3, *F*(1, 244) = 10.32, *p* =.002, η_p_^2^ =.039; Time 4, *F*(1, 244) = 4.91, *p* =.028, η_p_^2^ =.019. The difference between the nostalgia and control condition was no longer significant at Time 5, *F*(1, 244) = 0.67, *p* =.413, η_p_^2^ =.003.

We next probed the Induction Method × Time interaction. The induction method interacted with the quadratic trajectory over time only (Table 8). Tests of simple quadratic trajectories showed that this trajectory was significant in both the ERT condition, *F*(1, 244) = 120.18, *p* <.001, η_p_^2^ =.330, and VRT condition, *F*(1, 244) = 38.52, *p* <.001, η_p_^2^ =.136, but was stronger in the former than the latter condition. The drop in felt nostalgia between Time 1 and Time 2, as well as the subsequent leveling off, were most pronounced in the ERT (Fig. 4, left panel). In comparison, the decline in felt nostalgia over time was more gradual in the VRT (right panel). Tests of simple induction method effects revealed that felt nostalgia was higher in the ERT than VRT condition at Time 1, *F*(1, 244) = 4.79, *p* =.030, η_p_^2^ =.019, and Time 5, *F*(1, 244) = 7.40, *p* =.007, η_p_^2^ =.029. The induction-method effect was not significant at the three intermediate time points: Time 2, *F*(1, 244) = 1.41, *p* =.237, η_p_^2^ =.006; Time 3, *F*(1, 244) = 0.08, *p* =.780, η_p_^2^ <.001; Time 4, *F*(1, 244) = 1.51, *p* =.221, η_p_^2^ =.006.

The Nostalgia × Induction Method × Time three-way interaction was not significant (Table 7), indicating that the Nostalgia × Induction Method interaction effect (see above, under ‘Between-participant effects’) did not vary significantly in magnitude between time points. In other words, the VRT induction of nostalgia had a stronger impact on felt nostalgia than did the ERT induction and retained this advantage for the duration of the experiment (Fig. 4).^2^

##### Simple induction method effects by time point

When, in the analysis of between-participant effects, we probed the significant Nostalgia × Induction Method interaction, the simple induction method effect was significant in the control condition only, indicating that the greater effectiveness of the VRT (compared to ERT) was primarily attributable to lower felt nostalgia in the control-VRT than control-ERT condition. However, inspection of Fig. 4 suggests that this overall analysis may have obscured differences between time points. Specifically, at Times 2 and 3, the induction method appears to have a sizeable effect in the nostalgia condition. We evaluated this possibility by testing the effects of the simple induction method at each time point. At Time 1 (*F*[1, 244] = 9.58, *p* =.002, η_p_^2^ =.038) and Time 5 (*F*[1, 244] = 6.17, *p* =.014, η_p_^2^ =.020), the simple effect of induction method was significant in the control condition only; felt nostalgia was lower in the control-VRT than control-ERT condition. However, at Time 2 (*F*[1, 244] = 4.83, *p* =.029, η_p_^2^ =.019) and Time 3 (*F*[1, 244] = 4.38, *p* =.037, η_p_^2^ =.018), the simple effect of induction type was significant in the nostalgia condition only; felt nostalgia was higher in the nostalgia-VRT than nostalgia-ERT condition. The latter finding is important, showing that the VRT (compared to ERT) prolonged felt nostalgia in the nostalgia condition (rather than merely reducing felt nostalgia in the control condition).

#### Psychological benefits

##### Between-participant effects: Effects averaged across time points

**Fig. 5:**
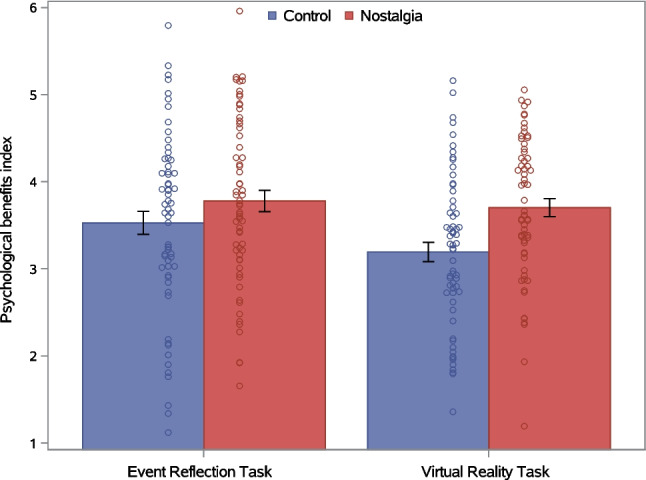

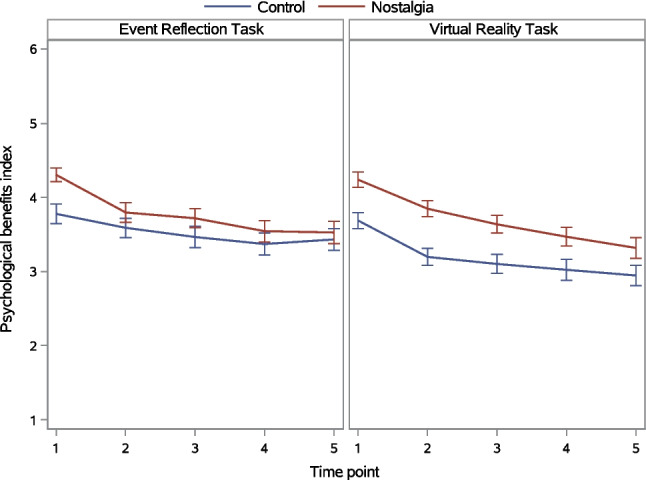
Psychological benefits as a function of nostalgia (vs. control) and induction method in Experiment 2. *Note. Error bars* represent standard errors

Consistent with H2, a significant main effect of nostalgia (vs. control) on the psychological benefits index showed that participants in the nostalgia condition reported higher overall psychological benefits than those in the control condition (Fig. 5). The main effect of induction type was not significant, nor was the Nostalgia × Induction Method interaction (Table 6). The absence of a significant interaction effect indicates that, contrary to H4, the VRT induction of nostalgia did not produce stronger psychological benefits than the ERT induction. Both the nostalgia and induction method main effects were qualified, however, by significant interactions with the within-participants effect of time.

##### Within-participant effects of time: Changes over time

**Fig. 6:**
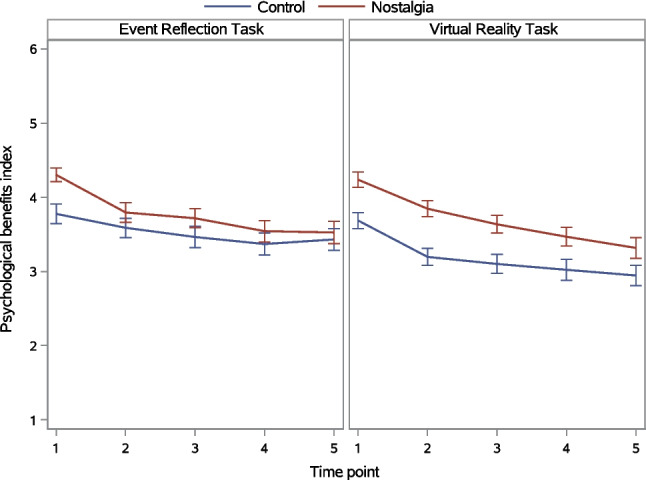
Psychological benefits index as a function of nostalgia, induction method, and time in Experiment 2. *Note. Error bars* represent standard errors

Figure 6 depicts changes in the psychological benefits index over time as a function of the nostalgia and induction method manipulations. A significant main effect of time indicated that psychological benefits differed between time points. This time main effect was qualified by significant Nostalgia × Time and Induction Method × Time interactions. The Nostalgia × Induction Method × Time three-way interaction was not significant (Table 7).

We first probed the Nostalgia × Time interaction. Nostalgia (vs. control) interacted with the linear trajectory over time only (Table 8). Tests of simple linear trajectories revealed that this trajectory was significant in both the control condition, *F*(1, 244) = 40.85, *p* <.001, η_p_^2^ =.143, and nostalgia condition, *F*(1, 244) = 100.76, *p* <.001, η_p_^2^ =.293, but was stronger in the latter than the former condition. In both conditions, but particularly in the nostalgia condition, psychological benefits declined as time progressed. Tests of simple nostalgia (vs. control) effects showed that participants in the nostalgia condition reported higher psychological benefits than those in the control condition at the first four time points and that this effect declined over time: Time 1, *F*(1, 244) = 24.18, *p* <.001, η_p_^2^ =.090; Time 2, *F*(1, 244) = 12.33, *p* <.001, η_p_^2^ =.047; Time 3, *F*(1, 244) = 9.20, *p* =.003 η_p_^2^ =.036; Time 4, *F*(1, 244) = 4.83, *p* =.029, η_p_^2^ =.019. The difference was no longer significant at Time 5, *F*(1, 244) = 2.64, *p* =.106, η_p_^2^ =.011.

Next, we probed the Induction Method × Time interaction. Induction method interacted with the linear trajectory only (Table 8). Tests of simple linear trajectories showed that this trajectory was significant in both the ERT condition, *F*(1, 244) = 45.86, *p* <.001, η_p_^2^ =.158, and VRT condition, *F*(1, 244) = 93.25, *p* <.001, η_p_^2^ =.277, but was stronger in the latter than the former condition. In both conditions, but particularly in the VRT condition, psychological benefits declined gradually as time progressed (Fig. 6, right panel). In comparison, the trajectory was flatter in the ERT condition (left panel). Tests of simple induction method effects revealed that participants completing the ERT reported higher psychological benefits than those completing the VRT at Time 5 only, *F*(1, 244) = 5.86, *p* =.016, η_p_^2^ =.023. The induction method effect was not significant at the four preceding time points, *F*s(1, 244) < 2.93, *p*s >.088, η_p_^2^ <.012.

##### Ancillary analyses of psychological benefits

**Fig. 7:**
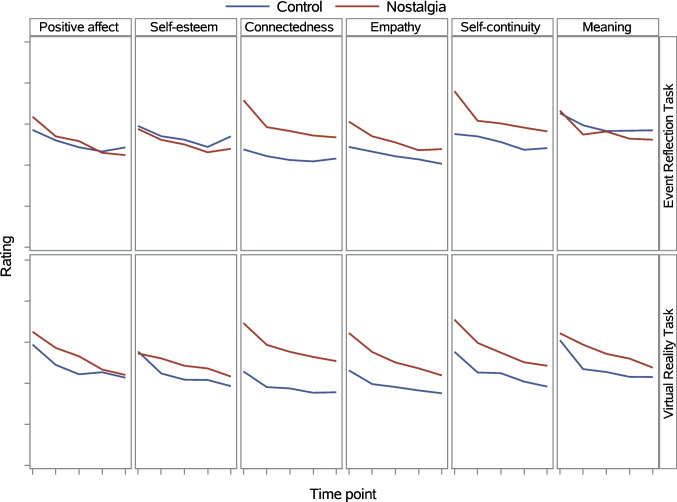
Disaggregated psychological benefits as a function of nostalgia, induction method, and time in Experiment 2

We depict in Fig. 7 the trajectories over time for each of the six psychological benefits as a function of nostalgia (vs. control) and induction method. The trajectories of social connectedness, empathy, and self-continuity were similar and generally matched the trajectory of the overall benefits index. To be precise, for each of these three psychological benefits, a significant nostalgia main effect was qualified by a Nostalgia × Time interaction effect (Tables 6, 8, 7), such that the difference between the nostalgia and control condition grew smaller over time. The trajectories of positive affect, self-esteem, and meaning showed a different pattern. We report detailed results for these three benefits in Supplemental Materials. We summarize the simple effects of nostalgia (vs. control) on positive affect, self-esteem, and meaning as follows: Nostalgia (compared to control) significantly increased positive affect at the first three time points. These effects were not qualified by the induction method. Nostalgia (compared to control) did not significantly increase (or decrease) self-esteem at any time point. Finally, in the VRT condition only, meaning in life was significantly higher in the nostalgia than control condition at Time 2. There was a trend in the same direction at Time 3 and Time 4.

#### Duration of nostalgia

We examined, exploratorily, the duration of nostalgic episodes. In the present context, the nostalgic episode starts with the experimental induction of nostalgia. The termination of the nostalgic episode, however, is more difficult to determine than the onset. Typically, an emotional episode is considered to have ended when emotion intensity returns to a baseline level (Verduyn, 2021; Verduyn et al., 2009). This raises the question of how to operationalize a return to baseline. One possibility is to treat the control condition as a baseline, such that the nostalgic episode terminates when felt nostalgia is no longer significantly higher in the nostalgia than the control condition. A problem with this approach is that different control conditions yield different baselines, introducing subjectivity. We followed an alternative approach and assumed that felt nostalgia has returned to baseline when it is no longer decreasing (i.e., its trajectory has leveled off). Accordingly, we ran a profile analysis, comparing felt nostalgia at adjacent time points (Table 9). In the nostalgia–ERT condition, felt nostalgia significantly decreased up to Time 3, but not thereafter. We therefore concluded that nostalgia had returned to baseline by Time 3 and thus lasted between 5 and 10 min. In the nostalgia–VRT condition, felt nostalgia significantly decreased at each time point. Assuming that it had returned to baseline by Time 5 (a reasonable assumption given the low level of nostalgia at that time point; *M* = 2.06), the average episode would have lasted between 15 and 20 min. These are conservative estimates, given that participants were distracted by filler tasks during the intervals between time points; distraction reduces emotion duration (Freund & Keil, 2013). The estimates are consistent with findings that emotional experiences in daily life typically last more than a few minutes (Verduyn, 2021) and underscore the value of the VRT.^3^

**Table 9:** Profile analysis comparing felt nostalgia at adjacent time points in Experiment 2

	Nostalgia-ERT condition			Nostalgia-VRT condition		
Felt nostalgia at:	*F*	*p*	η_p_^2^	*F*	*p*	η_p_^2^
Time 1 vs. Time 2	115.58	<.001	.321	69.29	<.001	.221
Time 2 vs. Time 3	4.76	.030	.019	5.92	.016	.024
Time 3 vs. Time 4	1.01	.316	.004	37.51	<.001	.133
Time 4 vs. Time 5	0.24	.628	<.001	13.60	<.001	.053

Degrees of freedom = 1, 244

### Discussion

The improved VRT induction of nostalgia bore fruit. Felt nostalgia was higher in the nostalgia than control condition (H1), and this effect was significantly larger in the VRT than ERT condition (H3; i.e., the Nostalgia × Induction Method interaction effect was significant). Importantly, the larger nostalgia effect in the VRT than ERT was durable (H5; i.e., the Nostalgia × Induction Method × Time three-way interaction was not significant). Although the greater efficacy of the VRT than ERT was due primarily to reductions in felt nostalgia in the control-VRT (compared to control-ERT) condition, felt nostalgia at Times 2 and 3 was significantly higher in the nostalgia-VRT (compared to nostalgia-ERT) condition. Indeed, whereas felt nostalgia decreased considerably between Time 1 and Time 2 in both the nostalgia-VRT and nostalgia-ERT conditions, the decline was more gradual in the former (Fig. 4, right panel) than the latter (left panel) condition. These findings attest to the VRT’s strength and are consistent with prior research demonstrating the capacity of virtual reality environments to evoke intense emotional experiences (Diniz Bernardo et al., 2021; Felnhofer et al., 2015).

The VRT yielded larger differences between the nostalgia and control conditions on felt nostalgia than did the ERT, yet this pattern did not carry over to psychological benefits, although felt nostalgia was significantly correlated with the benefits index at each time point (*r*s >.33, *p*s <.001). Instead, psychological benefits were significantly higher in the nostalgia than control condition (H2), irrespective of induction method (contrary to H4).^4^ The magnitude of this nostalgia effect on psychological benefits decreased linearly over time and was statistically significant up to Time 4 (15 min), with no indication that it was more durable in the VRT than ERT (contrary to H6).

Exploratory profile analyses gave initial insight into the duration of nostalgic episodes. These episodes lasted longer in the nostalgia-VRT (15–20 min) than the nostalgia-ERT condition (5–10 min). Estimates are conservative because participants were distracted by filler tasks and, in the nostalgia-VRT condition, the trajectory of felt nostalgia had not yet levelled off by the final time point.

### General discussion

In two experiments, we examined the potential of virtual reality environments to induce nostalgia. In Experiment 1, nostalgia (compared to control) increased felt nostalgia and conferred psychological benefits, irrespective of whether the emotion was induced with the established ERT or the VRT. These findings established the VRT’s viability but also showed that there was room for improvement. Accordingly, in Experiment 2, we fielded an improved version of the VRT. We introduced a birthday environment to give participants more choices, created a new Christmas environment with enhanced resolution and more decorations, augmented the playground environment with new equipment and avatars, and played scene-related sounds in all three environments. In addition, we measured felt nostalgia and psychological benefits repeatedly over a 20-min period. The virtual reality environments evoked vivid and durable feelings of nostalgia, more so than did the established ERT. That is, felt nostalgia was higher in the nostalgia (compared to control) condition, and this effect was larger in the improved VRT than ERT, remaining so over time. The VRT’s advantage was partly attributable to felt nostalgia generally being lower in the control–VRT than control–ERT condition. Yet, at Times 2 and 3, felt nostalgia was higher in the nostalgia–VRT than nostalgia–ERT condition, contributing to a temporal trajectory that was more gradual and took longer to return to baseline in the former than latter condition. These differences would have gone unnoticed, had we assessed felt nostalgia at a single time point only.

The VRT was not more effective than ERT in producing the beneficial effects of nostalgia (compared to control) or in extending these effects over time. Instead, psychological benefits were significantly higher in the nostalgia than control condition, with the magnitude of this difference diminishing gradually over time, irrespective of the induction method. Why did the induction method qualify the effect of nostalgia (compared to control) on felt nostalgia but not psychological benefits? To answer this question, it is helpful to think of a process or mediation model, with the nostalgia (vs. control) induction as independent variable. This independent variable increases felt nostalgia, the mediator. In turn, felt nostalgia leads to greater psychological benefits, the outcome. Kenny and Judd (2014) pointed out that mediators can be proximal or distal to the independent variable, and that some proximal mediators are essentially manipulation checks or measures of adherence. As felt nostalgia is essentially a manipulation check of the nostalgia (compared to control) induction, it can be considered a proximal mediator—it is “close to” the induction. By comparison, psychological benefits are more distal to the induction. This difference in proximity may explain why the relative strength of the VRT (compared to ERT) induction was more clearly reflected in felt nostalgia than in psychological benefits.

Although it is difficult to design virtual environments for evoking specific emotions (Felnhofer et al., 2015; Riva et al., 2007), the VRT proved highly effective. This new task expands the existing arsenal of inductions based on music (Sedikides et al., 2022), photographs (Yang et al., 2021), scents (Reid et al., 2015), and tastes (Reid et al., 2023), with strong potential for therapeutic application. For example, the VRT could be helpful for individuals with neurological conditions that impair the retrieval of nostalgic memories, such as stroke and dementia. Nostalgia confers important benefits to people living with dementia (Ismail et al., 2018, 2022), yet they progressively experience impaired autobiographical memory and, over time, may increasingly struggle to retrieve nostalgic memories. Inductions relying on autobiographical recall, such as the ERT, may therefore become less suitable. Virtual environments could aid people living with dementia through immersive, multisensory elicitation of nostalgia (Oliver et al., 2024a, 2024b). The VRT may also assist individuals with aphantasia, who experience impairments to visual imagery and, as a result, may find it difficult to generate visual images of past events (Keogh & Pearson, 2018; Monzel et al., 2023). The vivid virtual scenes could help them to experience nostalgia and its psychological benefits by compensating for visual-imagery deficits.

### Limitations and future directions

We note several limitations of our research. First, individuals’ involvement, immersion, and presence in the virtual environment can be increased further by augmenting scene realism, digital resolution, sound, and haptic feedback (Lamb et al., 2020; Witmer & Singer, 1998). In Experiment 2, we improved the VRT by adding animated avatars and scene-appropriate sound, yet the paradigm is still in its infancy. For example, follow-up studies could further enhance involvement, immersion, and presence by generating nostalgic virtual environments from omnidirectional (360°) camera captures of real-life environments (Kim et al., 2022). Such an approach would also enable researchers and practitioners to create bespoke, idiographic inductions tailored to specific individuals and their unique nostalgic memories (Oliver et al., 2024a, 2024b).

Second, we did not examine potential cultural differences in responses to the VRT. Although our experiments included some participants from non-Western backgrounds, they were not designed or sufficiently powered to explore cross-cultural variability in nostalgic experiences. This limits our ability to draw conclusions about the generalizability of the VRT beyond Western contexts. Given that nostalgia is shaped by culturally specific experiences, responses to nostalgic stimuli may vary across cultural groups (Abu-Rayya et al., 2025; Hepper et al., 2024; Wildschut et al., 2019). For example, whereas the Christmas scene successfully evoked nostalgia in our predominantly Western samples, its cultural specificity arguably limits generalizability. Future research could investigate how individuals from different cultural backgrounds engage with VR-based nostalgia inductions. Such studies should use tailored virtual scenes that are sensitive not only to cultural differences but also to variations in natural (e.g., flora, fauna) and built (e.g., architecture, furniture) environments.

Third, we explored, for the first time, the duration of nostalgic episodes. Our preliminary findings suggested that nostalgia lasted longer when induced with the VRT (15–20 min) than ERT (5–10 min). Might nostalgic episodes last even longer when induced with an idiographic induction based on omnidirectional (360°) camera captures of real-life environments? Further, emotion duration is negatively associated with distraction (Verduyn, 2021) and positively associated with duration of the precipitating event (Frijda, 2007). Might nostalgic episodes be extended by eliminating the distracting filler task and implementing a longer induction, rather than the brief inductions we used? Finally, emotional trajectories can be described in terms of three parameters (Verduyn et al., 2012): explosiveness (i.e., having high or low initial intensity), accumulation (i.e., peak intensity being located at the start or end of the episode), and reactivation (i.e., having a single or multiple intensity peaks). Our initial findings indicate that nostalgia is marked by high explosiveness, low accumulation, and low reactivation. This characterization is consistent with literary treatises on the emotion’s intense yet ephemeral nature (Kerouac, 1963; Proust, 1913) but needs replication.

Finally, in the VRT conditions of Experiments 1–2, participants not only immersed themselves in a virtual environment but were also instructed to recall an autobiographical memory related to that environment. Thus, those experiments did not present unequivocal evidence that being exposed to a nostalgic virtual environment is, in itself, sufficient to evoke nostalgia. To address this limitation, we ran a preregistered online experiment in which we showed participants video recordings of either the nostalgic or control virtual environments of Experiment 2 but did not instruct them to recall an autobiographical memory related to those environments. Those who were exposed to the nostalgic (compared to control) environments felt significantly more nostalgic (for details, see Supplemental Materials, Experiment S1).

The finding that mere exposure to nostalgic environments sufficed to elicit the emotion suggests that the VRT has notable potential future research: (a) the VRT could be used to induce nostalgia surreptitiously, thereby obviating the need for explicit recall instructions that may introduce demand characteristics; (b) the VRT could allow researchers to administer measures and tasks during, rather than after, the nostalgia induction, thereby removing a delay during which the emotion tends to dissipate; (c) the VRT harnesses sensory stimuli to evoke nostalgia, rather than requiring effortful autobiographical recall, thereby rendering it less vulnerable to participant inattentiveness or disengagement.

### Conclusion

Virtual reality environments can induce vivid, durable feelings of nostalgia and ensuing psychological benefits, with therapeutic potential for people living with cognitive impairments. Currently, in its infancy, we expect that rapid advances in the field of virtual reality and artificial intelligence will spawn increasingly immersive and cost-effective future implementations of this approach.

## Data Availability

https://osf.io/tn3pr/?view_only=59f788cbff354c2ab29b5516f2734cee

## Footnotes

1 Within the VRT condition, the distribution of participants across the four virtual environments was: Christmas scene (26.2%, *n* = 22), childhood scene (i.e., playground, 25.0%, *n* = 21), Christmas control (i.e., empty room; 22.6%, *n* = 19), and childhood control (i.e., empty playground, 26.2%, *n* = 22). We compared the level of nostalgia elicited by these four scenes. The Christmas scene (*M* = 5.30, *SD* = 1.63) and childhood scene (*M* = 5.38, *SD* = 1.12) did not evoke significantly different levels of nostalgia *(p* =.881). The Christmas control scene (*M* = 4.25, *SD* = 2.09) and childhood control scene (*M* = 3.50, *SD* = 1.92) did not evoke significantly different levels of nostalgia either (*p* =.165). Each of the two nostalgic scenes evoked significantly more nostalgia than either of the two control scenes (*p*s <.05).

2 In this context, “retention” can be operationalized in two ways. One can claim, as we did, that the VRT retained its advantage over the ERT for the entire duration of the experiment, because the Nostalgia Induction interaction did not vary significantly between time points. A stricter way to operationalize “retention” would be to ask for which time points the Nostalgia Induction interaction was statistically significant. When adopting this latter approach, the VRT retained its advantage over the ERT up to Time 3 (10 min).

3 This approach approximates emotion duration but we acknowledge that it confounds duration with statistical power, such that a given difference between time points could be statistically significant in a high-powered study but not in a low-powered one. To address this issue, researchers could specify a minimum effect size of interest (Lakens et al., 2018). However, this method introduces subjectivity (i.e., what should be the size of this minimum effect of interest?) and is beyond the scope of the present research.

4 Inspection of Fig. 6 suggests that, descriptively, the effect of nostalgia (compared to control) on the benefits index was larger in the VRT than ERT condition. This numerical difference was due to lower index scores in the VRT–control than ERT–control condition (as opposed to higher scores in the VRT–nostalgia than ERT–nostalgia condition).

## References

[CR1] Abu-Rayya, H. N., Abumuhaisen, Y., Wildschut, T., & Sedikides, C. (2025). Nostalgia in the Gaza Strip: Psychological costs and benefits of nostalgia among Palestinian youth. *British Journal of Social Psychology,**64*, e12859. 10.1111/bjso.12859 40130943 10.1111/bjso.12859 PMC11934847

[CR2] Baños, R. M., Botella, C., Rubió, I., Quero, S., García-Palacios, A., & Alcañiz, M. (2008). Presence and emotions in virtual environments: The influence of stereoscopy. *Cyber Psychology & Behavior,**11*(1), 1–8. 10.1089/cpb.2007.9936 10.1089/cpb.2007.9936 18275306

[CR3] Batcho, K. I. (1995). Nostalgia: A psychological perspective. *Perceptual and Motor Skills,**80*(1), 131–143. 10.2466/pms.1995.80.1.131 7624184 10.2466/pms.1995.80.1.131

[CR4] Batson, C. D., Fultz, J., & Schoenrade, P. A. (1987). Distress and empathy: Two qualitatively distinct vicarious emotions with different motivational consequences. *Journal of Personality,**55*(1), 19–39. 10.1111/j.1467-6494.1987.tb00426.x 3572705 10.1111/j.1467-6494.1987.tb00426.x

[CR5] Cheung, W.-Y., Wildschut, T., Sedikides, C., Hepper, E. G., Arndt, J., & Vingerhoets, A. J. J. M. (2013). Back to the future: Nostalgia increases optimism. *Personality and Social Psychology Bulletin,**39*(11), 1484–1496. 10.1177/0146167213499187 23928397 10.1177/0146167213499187

[CR6] Chirico, A., Ferrise, F., Cordella, L., & Gaggioli, A. (2018). Designing awe in virtual reality: An experimental study. *Frontiers in Psychology,**8*, Article 2351. 10.3389/fpsyg.2017.02351 29403409 10.3389/fpsyg.2017.02351 PMC5786556

[CR7] Davis, M. H. (1979). A multidimensional approach to individual differences in empathy. *JSAS Catalog of Selected Documents in Psychology,**10*, 85.

[CR8] Diemer, J., Alpers, G. W., Peperkorn, H. M., Shiban, Y., & Mühlberger, A. (2015). The impact of perception and presence on emotional reactions: A review of research in virtual reality. *Frontiers in Psychology,**6*, 26. 10.3389/fpsyg.2015.00026 25688218 10.3389/fpsyg.2015.00026 PMC4311610

[CR9] Diniz Bernardo, P., Bains, A., Westwood, S., & Mograbi, D. C. (2021). Mood induction using virtual reality: A systematic review of recent findings. *Journal of Technology in Behavioral Science,**6*(1), 3–24. 10.1007/s41347-020-00152-9

[CR10] Evans, N. D., Reyes, J., Wildschut, T., Sedikides, C., & Fetterman, A. K. (2021). Mental transportation mediates nostalgia’s psychological benefits. *Cognition and Emotion,**35*(1), 84–95. 10.1080/02699931.2020.1806788 32787551 10.1080/02699931.2020.1806788

[CR11] Felnhofer, A., Kothgassner, O. D., Schmidt, M., Heinzle, A. K., Beutl, L., Hlavacs, H., & Kryspin-Exner, I. (2015). Is virtual reality emotionally arousing? Investigating five emotion-inducing virtual park scenarios. *International Journal of Human-Computer Studies,**82*, 48–56. 10.1016/j.ijhcs.2015.05.004

[CR12] Fetterman, A. K., Evans, N. D., Ravey, E. P., Henderson, P. R., Tran, B. H. L., & Boyd, R. L. (2025). The topics of nostalgic recall: The benefits of nostalgia depend on the topics that one recalls. *Social Psychological and Personality Science,**16*(4), 444–456. 10.1177/19485506241229305

[CR13] Freund, A. M., & Keil, A. (2013). Out of mind, out of heart: Attention affects duration of emotional experience. *Cognition and Emotion,**27*(3), 549–557. 10.1080/02699931.2012.725574 22963519 10.1080/02699931.2012.725574

[CR14] Frijda, N. H. (2007). *The laws of emotion*. Erlbaum. 10.1037//0003-066x.43.5.349 3389582

[CR15] Hepper, E. G., Ritchie, T. D., Sedikides, C., & Wildschut, T. (2012). Odyssey’s end: Lay conceptions of nostalgia reflect its original Homeric meaning. *Emotion,**12*(1), 102–119. 10.1037/a0025167 21859192 10.1037/a0025167

[CR16] Hepper, E. G., Wildschut, T., Sedikides, C., Ritchie, T. D., Yung, Y.-F., Hansen, N., Abakoumkin, G., Arikan, G., Cisek, S. Z., Demassosso, D. B., Gebauer, J. E., Gerber, J. P., González, R., Kusumi, T., Misra, G., Rusu, M., Ryan, O., Stephan, E., Vingerhoets, A. J. J., & Zhou, X. (2014). Pancultural nostalgia: Prototypical conceptions across cultures. *Emotion,**14*(4), 733–747. 10.1037/a0036790 24866530 10.1037/a0036790

[CR17] Hepper, E. G., Wildschut, T., Sedikides, C., Robertson, S., & Routledge, C. D. (2021). Time capsule: Nostalgia shields psychological well-being from limited time horizons. *Emotion,**21*(3), 644–664. 10.1037/emo0000728 32191101 10.1037/emo0000728

[CR18] Hepper, E. G., Sedikides, C., Wildschut, T., Cheung, W. Y., Abakoumkin, G., Arikan, G., Aveyard, M., Baldursson, E. B., Bialobrzeska, O., Bouamama, S., Bouzaouech, I., Brambilla, M., Burger, A. M., Chen, S. X., Cisek, S., Demassosso, D., Estevan-Reina, L., González Gutiérrez, R., Gu, L., & Zengel, B. (2024). Pancultural nostalgia in action: Prevalence, triggers, and psychological functions of nostalgia across cultures. *Journal of Experimental Psychology: General,**153*(3), 754–778. 10.1037/xge0001521 38252088 10.1037/xge0001521

[CR19] Holak, S. L., & Havlena, W. J. (1992). Nostalgia: An exploratory study of themes and emotions in the nostalgic experience. *Advances in Consumer Research,**19*, 380–387.

[CR20] Ismail, S., Christopher, G., Dodd, E., Wildschut, T., Sedikides, C., Ingram, T., Jones, R. W., Noonan, K. A., Tingley, D., & Cheston, R. (2018). Psychological and mnemonic benefits of nostalgia for people with dementia. *Journal of Alzheimer’s Disease,**65*, 1327–1344. 10.3233/JAD-180075 30149444 10.3233/JAD-180075

[CR21] Ismail, S., Dodd, E., Christopher, G., Wildschut, T., Sedikides, C., & Cheston, R. (2022). The content and function of nostalgic memories of people living with dementia. *The International Journal of Aging and Human Development,**94*(4), 436–459. 10.1177/00914150211024185 34110940 10.1177/00914150211024185 PMC8958641

[CR22] Joseph, D. L., Chan, M. Y., Heintzelman, S. J., Tay, L., Diener, E., & Scotney, V. S. (2020). The manipulation of affect: A meta-analysis of affect induction procedures. *Psychological Bulletin,**146*(4), 355–375. 10.1037/bul0000224 31971408 10.1037/bul0000224

[CR23] Juhl, J., Wildschut, T., Sedikides, C., Diebel, T., Cheung, W.-Y., & Vingerhoets, A. J. J. M. (2020). Nostalgia proneness and empathy: Generality, underlying mechanism, and implications for prosocial behavior. *Journal of Personality,**88*(3), 485–500. 10.1111/jopy.12505 31442311 10.1111/jopy.12505

[CR24] Juhl, J., & Biskas, M. (2023). Nostalgia: An impactful social emotion. *Current Opinion in Psychology,**49*, Article 101545. 10.1016/j.copsyc.2022.101545 36641833 10.1016/j.copsyc.2022.101545

[CR25] Kenny, D. A., & Judd, C. M. (2014). Power anomalies in testing mediation. *Psychological Science,**25*(2), 334–339. 10.1177/0956797613502676 24311476 10.1177/0956797613502676

[CR26] Keogh, R., & Pearson, J. (2018). The blind mind: No sensory visual imagery in aphantasia. *Cortex,**105*, 53–60. 10.1016/j.cortex.2017.10.012 29175093 10.1016/j.cortex.2017.10.012

[CR27] Kerouac, J. (1963). *Visions of Gerard*. Farrar.

[CR28] Kim, H., Remaggi, L., Dourado, A., Campos, T. D., Jackson, P. J., & Hilton, A. (2022). Immersive audio-visual scene reproduction using semantic scene reconstruction from 360 cameras. *Virtual Reality,**26*, 823–838. 10.1007/s10055-021-00594-3

[CR29] Kuppens, P., & Verduyn, P. (2017). Emotion dynamics. *Current Opinion in Psychology,**17*, 22–26. 10.1016/j.copsyc.2017.06.004 28950968 10.1016/j.copsyc.2017.06.004

[CR30] Lakens, D., Scheel, A. M., & Isager, P. M. (2018). Equivalence testing for psychological research: A tutorial. *Advances in Methods and Practices in Psychological Science,**1*(2), 259–269. 10.1177/2515245918770963

[CR31] Lamb, R., Lin, J., & Firestone, J. B. (2020). Virtual reality laboratories: A way forward for schools? *EURASIA Journal of Mathematics, Science and Technology Education,**16*(6), Article em1856. 10.29333/ejmste/8206

[CR32] Leunissen, J. M. (2023). Diamonds and rust: The affective ambivalence of nostalgia. *Current Opinion in Psychology,**49*, Article 101541. 10.1016/j.copsyc.2022.101541 36608387 10.1016/j.copsyc.2022.101541

[CR33] Leunissen, J. M., Wildschut, T., Sedikides, C., & Routledge, C. (2021). The hedonic character of nostalgia: An integrative data analysis. *Emotion Review,**13*(2), 139–156. 10.1177/1754073920950455

[CR34] Monzel, M., Agren, T., Tengler, M., & Reuter, M. (2023). Imaginal extinction without imagery: Dissociating the effects of visual imagery and propositional thought by contrasting participants with aphantasia, simulated aphantasia, and controls. *Psychophysiology,**00*, Article e14271. 10.1111/psyp.14271 10.1111/psyp.14271 36762753

[CR35] Morie, J. F. (2006). Virtual reality, immersion, and the unforgettable experience. *Stereoscopic Displays and Virtual Reality Systems XIII,**6055*, 621–630. 10.1117/12.660290

[CR36] Oliver, A., Wildschut, T., Redhead, E., Parker, M., Saif, S., Wood, A., Sedikides, C., & Cheston, R. (2024a). Benefits of nostalgic landmarks for people living with Alzheimer’s disease. *Journal of Alzheimer’s Disease,**102*(3), 683–702. 10.1177/13872877241291908 39670735 10.1177/13872877241291908

[CR37] Oliver, A., Wildschut, T., Sedikides, C., Parker, M. O., Wood, A. P., & Redhead, E. S. (2024b). Nostalgia assuages spatial anxiety. *Journal of Experimental Social Psychology,**112*, Article 104586. 10.1016/j.jesp.2023.104586

[CR38] Proust, M. (1913). *Du côté de chez Swann.* Grasset.

[CR39] Reid, C. A., Green, J. D., Wildschut, T., & Sedikides, C. (2015). Scent-evoked nostalgia. *Memory,**23*(2), 157–166. 10.1080/09658211.2013.876048 24456210 10.1080/09658211.2013.876048

[CR40] Reid, C. A., Green, J. D., Buchmaier, S., McSween, D. K., Wildschut, T., & Sedikides, C. (2023). Food-evoked nostalgia. *Cognition and Emotion,**37*(1), 34–48. 10.1080/02699931.2022.2142525 36331076 10.1080/02699931.2022.2142525

[CR41] Riva, G., Mantovani, F., Capideville, C. S., Preziosa, A., Morganti, F., Villani, D., Gaggioli, A., Botella, C., & Alcañiz, M. (2007). Affective interactions using virtual reality: The link between presence and emotions. *Cyberpsychology & Behavior,**10*(1), 45–56. 10.1089/cpb.2006.9993 17305448 10.1089/cpb.2006.9993

[CR42] Routledge, C., Wildschut, T., Sedikides, C., & Juhl, J. (2013). Nostalgia as a resource for psychological health and well-being. *Social and Personality Psychology Compass,**7*(11), 808–818. 10.1111/spc3.12070

[CR43] Sedikides, C., Leunissen, J. M., & Wildschut, T. (2022). The psychological benefits of music-evoked nostalgia. *Psychology of Music,**50*(6), 2044–2062. 10.1177/03057356211064641

[CR44] Sedikides, C., & Wildschut, T. (2018). Finding meaning in nostalgia. *Review Of General Psychology,**22*(1), 48–61. 10.1037/gpr0000109

[CR45] Sedikides, C., & Wildschut, T. (2019). The sociality of personal and collective nostalgia. *European Review of Social Psychology,**30*(1), 123–173. 10.1080/10463283.2019.1630098

[CR46] Sedikides, C., & Wildschut, T. (2025). From self-discontinuity to meaning through nostalgia. In K. Fujita, A. Fishbach, & N. Liberman (Eds.), *The psychological quest for meaning* (pp. 231–253). Guilford Press.

[CR47] Sedikides, C., Wildschut, T., Arndt, J., & Routledge, C. (2008). Nostalgia: Past, present, and future. *Current Directions in Psychological Science,**17*(5), 304–307. 10.1111/j.1467-8721.2008.00595.x

[CR48] Sedikides, C., Wildschut, T., Cheung, W. Y., Routledge, C., Hepper, E. G., Arndt, J., Vail, K., Zhou, X., Brackstone, K., & Vingerhoets, A. J. (2016). Nostalgia fosters self-continuity: Uncovering the mechanism (social connectedness) and consequence (eudaimonic well-being). *Emotion,**16*(4), 524–539. 10.1037/emo0000136 26751632 10.1037/emo0000136

[CR49] Sedikides, C., Wildschut, T., Conway, P., & Lasaleta, J. (2025). Nostalgia as a moral emotion. In S. Laham (Ed.), *Handbook of ethics and social psychology* (pp. 158–167). Edward Elgar Publishing. 10.4337/9781035311804.00021

[CR50] Sedikides, C., Wildschut, T., Routledge, C., & Arndt, J. (2015a). Nostalgia counteracts self-discontinuity and restores self-continuity. *European Journal of Social Psychology,**45*(1), 52–61. 10.1002/ejsp.2073

[CR51] Sedikides, C., Wildschut, T., Routledge, C., Arndt, J., Hepper, E. G., & Zhou, X. (2015). To nostalgize: Mixing memory with affect and desire. *Advances in experimental social psychology* (Vol. 51, pp. 189–273). Academic Press. 10.1016/bs.aesp.2014.10.001

[CR52] Shadish, W. R., Cook, T. D., & Campbell, D.T. (2002). *Experimental and quasi-experimental designs for generalized causal inference.* Houghton Mifflin.

[CR53] The New Oxford Dictionary of English. (1998). Oxford University Press.

[CR54] Thomson, N. D., Aboutanos, M., Kiehl, K. A., Neumann, C., Galusha, C., & Fanti, K. A. (2019). Physiological reactivity in response to a fear-induced virtual reality experience: Associations with psychopathic traits. *Psychophysiology,**56*(1), Article e13276. 10.1111/psyp.13276 30129671 10.1111/psyp.13276

[CR55] Unity Technologies. (2018). *Unity real-time development platform | 3D, 2D, VR & AR*. Unity. https://unity.com/

[CR56] Van Tilburg, W. A. P., Wildschut, T., & Sedikides, C. (2018). Nostalgia’s place among self-conscious emotions. *Cognition and Emotion,**32*(4), 742–759. 10.1080/02699931.2017.1351331 28738756 10.1080/02699931.2017.1351331

[CR57] Verduyn, P. (2021). Emotion duration. In C. E. Waugh & P. Kuppens (Eds.), *Affect dynamics* (pp. 3–18). Springer Nature Switzerland AG. 10.1007/978-3-030-82965-0_1

[CR58] Verduyn, P., Delaveau, P., Rotgé, J.-Y., Fossati, P., & van Mechelen, I. (2015). Determinants of emotion duration and underlying psychological and neural mechanisms. *Emotion Review,**7*(4), 330–335. 10.1177/1754073915590618

[CR59] Verduyn, P., Delvaux, E., van Coillie, H., Tuerlinckx, F., & van Mechelen, I. (2009). Predicting the duration of emotional experience: Two experience sampling studies. *Emotion,**9*(1), 83–91. 10.1037/a0014610 19186919 10.1037/a0014610

[CR60] Verduyn, P., Van Mechelen, I., & Frederix, E. (2012). Determinants of the shape of emotion intensity profiles. *Cognition And Emotion,**26*(8), 1486–1495. 10.1080/02699931.2012.662152 22360656 10.1080/02699931.2012.662152

[CR61] Vess, M., Arndt, J., Routledge, C., Sedikides, C., & Wildschut, T. (2012). Nostalgia as a resource for the self. *Self and Identity,**11*(3), 273–284. 10.1080/15298868.2010.521452

[CR62] Waugh, C. E., Hamilton, J. P., & Gotlib, I. H. (2010). The neural temporal dynamics of the intensity of emotional experience. *Neuroimage,**49*(2), 1699–1707. 10.1016/j.neuroimage.2009.10.006 19833213 10.1016/j.neuroimage.2009.10.006 PMC2794551

[CR63] Wildschut, T., & Sedikides, C. (2025). Psychology and nostalgia: A primer on experimental nostalgia inductions. In T. Becker & D. Trigg (Eds.), *The Routledge handbook of nostalgia* (pp. 54–69). Routledge.

[CR64] Wildschut, T., Sedikides, C., & Alowidy, D. (2019). *Hanin*: Nostalgia among Syrian refugees. *European Journal of Social Psychology,**49*(7), 1368–1384. 10.1002/ejsp.2590

[CR65] Wildschut, T., Sedikides, C., Arndt, J., & Routledge, C. (2006). Nostalgia: Content, triggers, functions. *Journal of Personality and Social Psychology,**91*(5), 975–993. 10.1037/0022-3514.91.5.975 17059314 10.1037/0022-3514.91.5.975

[CR66] Witmer, B. G., & Singer, M. J. (1998). Measuring presence in virtual environments: A presence questionnaire. *Presence: Teleoperators and Virtual Environments,**7*(3), 225–240. 10.1162/105474698565686

[CR67] Yang, Z., Sedikides, C., Izuma, K., Wildschut, T., Kashima, E. S., Luo, Y., Chen, J., & Cai, H. (2021). Nostalgia enhances detection of death threat: Neural and behavioral evidence. *Scientific Reports,**11*(1), Article 12662. 10.1038/s41598-021-91322-z 34135348 10.1038/s41598-021-91322-z PMC8209061

